# Mechanistic and Predictive QSAR Analysis of Diverse Molecules to Capture Salient and Hidden Pharmacophores for Anti-Thrombotic Activity

**DOI:** 10.3390/ijms22158352

**Published:** 2021-08-03

**Authors:** Magdi E. A. Zaki, Sami A. Al-Hussain, Vijay H. Masand, Manoj K. Sabnani, Abdul Samad

**Affiliations:** 1 Department of Chemistry, Faculty of Science, Imam Mohammad Ibn Saud Islamic University, Riyadh 13318, Saudi Arabia; sahussain@imamu.edu.sa; 2 Department of Chemistry, Vidya Bharati Mahavidyalaya, Amravati 444 601, Maharashtra, India; 3 Department of Biology, The University of Texas at Arlington, Arlington, TX 76019, USA; manojkumar.sabnani@mavs.uta.edu; 4 Department of Pharmaceutical Chemistry, Faculty of Pharmacy, Tishk International University, Erbil 44001, Kurdistan Region, Iraq; abdul.samad@tiu.edu.iq

**Keywords:** thrombosis, factor Xa, QSAR, machine learning, pharmacophores

## Abstract

Thrombosis is a life-threatening disease with a high mortality rate in many countries. Even though anti-thrombotic drugs are available, their serious side effects compel the search for safer drugs. In search of a safer anti-thrombotic drug, Quantitative Structure-Activity Relationship (QSAR) could be useful to identify crucial pharmacophoric features. The present work is based on a larger data set comprising 1121 diverse compounds to develop a QSAR model having a balance of acceptable predictive ability (Predictive QSAR) and mechanistic interpretation (Mechanistic QSAR). The developed six parametric model fulfils the recommended values for internal and external validation along with Y-randomization parameters such as R^2^_tr_ = 0.831, Q^2^_LMO_ = 0.828, R^2^_ex_ = 0.783. The present analysis reveals that anti-thrombotic activity is found to be correlated with concealed structural traits such as positively charged ring carbon atoms, specific combination of aromatic Nitrogen and sp2-hybridized carbon atoms, etc. Thus, the model captured reported as well as novel pharmacophoric features. The results of QSAR analysis are further vindicated by reported crystal structures of compounds with factor Xa. The analysis led to the identification of useful novel pharmacophoric features, which could be used for future optimization of lead compounds.

## 1. Introduction

World Thrombosis Day (WTD) is celebrated on 13 October each year in memory of Rudolf Virchow, who developed the concept of “thrombosis”. Thrombosis, which is responsible for high mortality in the U.S. and Europe, involves the formation of pathologically dangerous clots in the artery or vein [1]. Recent studies point out that COVID-19 or vaccines approved to fight against COVID-19 could lead to the formation of clots [2,3,4,5,6]. The herpes simplex virus type-1 surface is responsible for the initiation of thrombus formation [7]. For cancer patients, thrombosis substantially decreases the survival rate [8,9]. The main reasons for thrombosis include age, surgery, trauma, inflammation, cancer, vessel injury, or overexpression of thrombogenic factors, to mention a few [1,8,9,10,11,12,13]. The understanding of thrombus development and its inhibition has gained a high interest to develop a safer and orally active anticoagulant for the treatment and prevention of thrombotic diseases. The cascade of thrombus development involves a good number of enzymes like factor X, prothrombin, thrombin, etc. [1,8,9,10,11,12,13].

**Figure 1:**
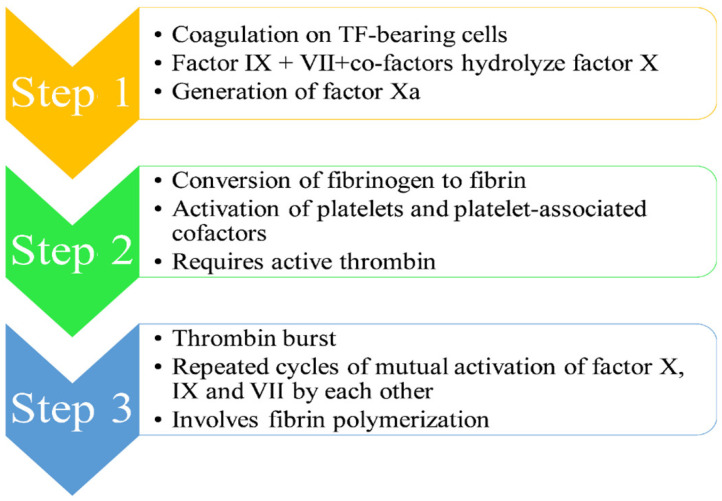
Depiction of mechanism of thrombus formation.

Stuart–Prower factor, or factor X, is a vitamin K-dependent enzyme (EC 3.4.21.6) synthesized in the liver [1,8,9,10,13]. It is a serine protease with a half-life of 40–45 h and acts as the first enzyme in the coagulation cascade, consequently making it essential for the thrombin pathway. The cell-based model of anticoagulation identifies three main stages (see Figure 1) [1,8,9,10,13]:

(1) Step 1 [1,8,9,10,11,13]: The mechanism of the coagulation cascade begins with coagulation on TF-bearing cells. Factor IX and VII, along with their respective co-factors, are responsible for the hydrolysis of factor X, leading to its conversion to its activated form Xa. The activated factor Xa is accountable for the dual breaking of prothrombin first at an arg-thr and then at an arg-ile bond, thereby generating active thrombin, which is a coagulation protease. A single factor X converts several prothrombin molecules, thus generating multiple thrombin molecules.
(2) Step 2 [1,8,9,10,11,13]: The second step involves conversion of fibrinogen to fibrin, which is accomplished by the activation of platelets and platelet-associated cofactors in the presence of a sufficient quantity of active thrombin.
(3) Step 3 [1,8,9,10,11,13]: The third step involves “thrombin burst”, which occurs due to continuous generation of thrombin on the platelet surface, thereby leading to repeated cycles of mutual activation of factor X, IX and VII by each other. This thrombin burst through fibrin polymerization is vital for a thrombus formation.

Factor Xa has been identified to play a significant involvement in all three stages [8,9,10,13]. Additionally, it bridges the intrinsic and extrinsic pathways to the common coagulation pathway, which makes it a legitimate target to block the activation cascade of the thrombus formation [1]. Thus, inhibition of factor Xa will reduce the development of new thrombin without disturbing the minimal thrombin level required for primary hemostasis. Therefore, many marketed anti-coagulating agents like Warfarin, Phenprocoumon, Acenocoumarol, Rivaroxaban, etc. [14] act either by inhibiting the synthesis of factor Xa or its activity. 

**Figure 2:**
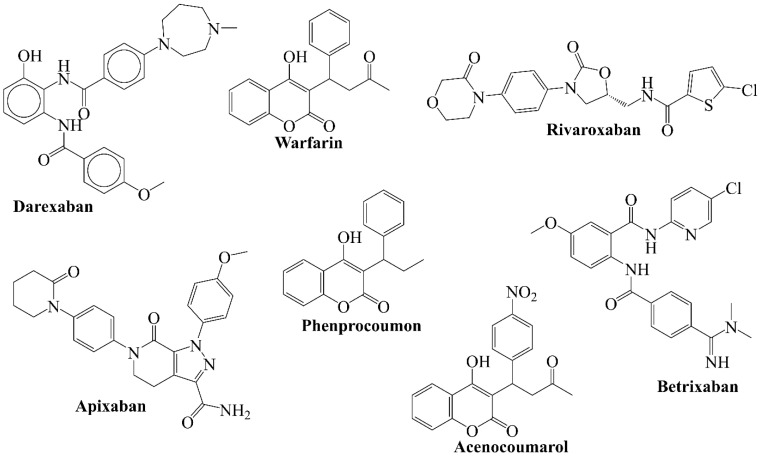
Some marketed anti-coagulating drugs.

Despite the availability of many different marketed drugs (see Figure 2) [1,10,12,14,15], the high mortality and associated side effects like bleeding, spinal hematoma, anaphylaxis along with a high necessity of continuous monitoring of patients indicate that there is a need for a better anticoagulant [1,9,10,12,14,15]. While optimizing the ADMET profile of a compound, it necessary to retain the features associated with high activity (pharmacophoric features). Therefore, a rational analysis of different anti-coagulating agents is required to recognize prominent and visually unrecognizable pharmacophoric features. To achieve this goal, there is a prerequisite to perform computer-assisted analysis like QSAR, virtual screening, etc., of a larger dataset of anti-coagulating agents. A rational drug designing approach such as QSAR is a method of choice due to a good number of advantages, including cost reduction, minimal trial and error, its time efficient nature, etc. [16,17,18,19] A typical QSAR analysis is a machine learning approach, which involves a systematic approach which begins with selection of a dataset followed by its methodical analysis to identify pharmacophoric features (Mechanistic/Descriptive QSAR) and to predict the activity of a compound before its wet lab synthesis and biological testing (Predictive QSAR) [16,17,19,20].

Different researchers have reported QSAR models for factor Xa. For example, Matter et al. [21] used a dataset of 3-Oxybenzamides (107 molecules) to perform 3D-QSAR, which had acceptable statistical performance with R^2^_tr_ = 0.95 and Q^2^ = 0.74. Ye et al. [21] performed QSAR using Thiophene-anthranilamides. However, use of smaller data sets comprised of molecules with fewer scaffolds/pharmacophoric features thereby limited applicability of those QSAR models and confined their use in optimization to a few classes of compounds [14,21,22]. A QSAR analysis based on a larger dataset comprised of diverse scaffolds with a balance of acceptable predictive capability and mechanistic interpretations is highly beneficial for lead optimization. Therefore, the present work involves QSAR analysis of a dataset comprised of a large number of diverse anti-coagulating agents. The results could be useful to develop a novel compound as an anti-coagulating agent. 

## 2. Results

The present QSAR analysis was performed using a large dataset comprised of structurally diverse compounds with experimentally measured Ki in the range between 0.007 to 18,000 nM. Thus, it covers a sufficiently broad chemical and data range. This helped to derive a properly validated [19,23,24,25,26,27] genetic algorithm unified with a multilinear regression (GA-MLR) model to collect or extend thorough information about the pharmacophoric features that control a desired bio-activity (Descriptive QSAR) while having adequate external predictive capability (Predictive QSAR). The six variable-based GA-MLR QSAR model (see Equation (1)), along with selected internal and external validation parameters (see Supplementary Material for additional parameters), is as follows:

pKi = 6.176 (±0.073) + 1.513 (±0.104) * ringCplus_sumpc + 0.519 (±0.04) * aroN_sp2C_4B + 1.197 (±0.077) * fClamdN5B − 1.018 (±0.099) * fsp2Osp3O6B − 1.091 (±0.111) * fsp2Nsp3O9B − 0.9 (±0.158) * fsp2Csp2O8B R^2^_tr_ = 0.831, R^2^_adj._ = 0.83, RMSE_tr_ = 0.476, CCC_tr_ = 0.908, s = 0.478, F = 731.048, R^2^_cv_ (Q^2^loo) = 0.829, RMSE_cv_ = 0.479, CCC_cv_ = 0.907, Q^2^_LMO_ = 0.828, R^2^_Yscr_ = 0.007, RMSE_ex_ = 0.526, R^2^_ex_ = 0.783, Q^2^ − F^1^ = 0.782, Q^2^ − F^2^ = 0.782, Q^2^ − F^3^ = 0.794, CCC_ex_ = 0.874, R^2^ − ExPy = 0.783, R′_o_^2^ = 0.704, k′ = 0.996, 1 − (R^2^/R′_o_^2^) = 0.101, R_o_^2^ = 0.782, k = 0.999, 1 − (R^2^ − ExPy/R_o_^2^) = 0.001 (1)

**Figure 3:**
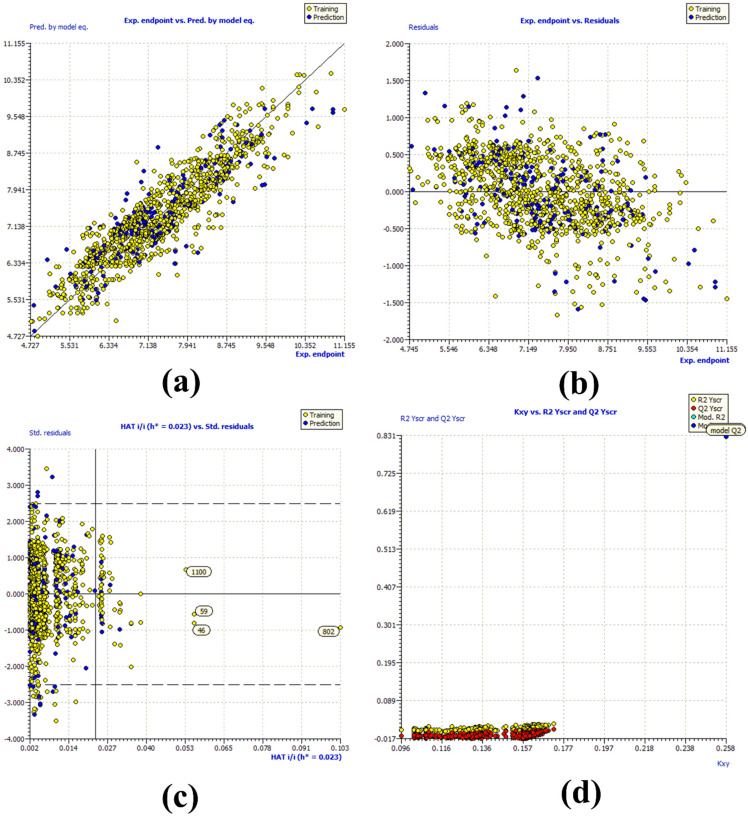
Graph for (**a**) experimental vs. predicted pKi (the solid line represents the regression line); (**b**) experimental vs. residuals; (**c**) Williams plot for applicability domain (the vertical solid line represents h* = 0.023 and horizontal dashed lines represent the upper and lower boundaries for applicability domain); (**d**) Y-randomization.

The above statistical validation parameters are recommended to judge the internal and external robustness and have the usual meaning (see Supplementary Material for detailed descriptions and formulae). The high value of different parameters like R^2^_tr_ (coefficient of determination), R^2^_adj._ (adjusted coefficient of determination), and R^2^_cv_ (Q^2^loo) (cross-validated coefficient of determination for leave-one-out), R^2^_ex_ (external coefficient of determination), Q^2^ − F^n^ and CCC_ex_ (concordance correlation coefficient) etc. and low value of LOF (lack-of-fit), RMSE_tr_ (root mean square error), MAE_tr_ (mean absolute error), R^2^_Yscr_ (R^2^ for Y-scrambling), etc. along with the different graphs related with the model point out that the model is statistically robust with excellent internal and external predictive ability as well as free from chancy correlation [19,23,24,25,26,27,28,29,30]. Moreover, the Williams plot indicates that the model is statistically acceptable (see Figure 3) [16,19,26,29,30,31]. Therefore, it fulfils all the Organisation for Economic Co-operation and Development (OECD) recommended guidelines for creating a useful QSAR model.

## 3. Discussion

### 3.1. Mechanistic Interpretation of QSAR Model

A properly validated correlation between salient features of the molecules, represented by molecular descriptors, and their bioactivity expands information about mechanistic aspects of molecules, specificity and quantity (presence and even absence) of various structural traits for the desired bioactivity. In the present analysis we have compared the Ki values of different molecules in correlation and as an effect of a specific molecular descriptor; however, an analogous or opposite effect of other molecular descriptors or unknown factors having a dominant effect in determining the overall Ki value of a molecule cannot be neglected. In other words, a single molecular descriptor is incapable of fully explaining the experimental Ki value for such a diverse set of molecules. That is, the successful utilization of the developed QSAR model relies on the concomitant use of constituent molecular descriptors.

The molecular descriptor **ringCplus_sumpc** stands for the sum of partial charges on positively charged ring carbon atoms. It has a positive coefficient in the QSAR model; therefore, augmenting its value could result in improved activity against factor Xa. From this, it appears that mere ring carbon atoms or only positively charged carbon atoms are independently very important, but replacing **ringCplus_sumpc** by **ringC** (number of ring carbon atoms) or **nCplus** (number of positively charged carbon atoms) significantly reduced the statistical performance of the model (R^2^ = 0.68–0.71). Similarly, replacement of **ringCplus_sumpc** by **naroC** (number of aromatic carbon atoms) and **naroCplus** (number of positively charged aromatic carbon atoms) resulted in reduced statistical performance of the model (R^2^ = 0.68–0.75). Moreover, **ringCplus_sumpc,** has a better correlation with pKi than **ringC, nCplus**, **naroC** and **naroCplus** (see Supplementary Material). Therefore, merely increasing the number of ring or aromatic carbon atoms is not sufficient. It is essential to increase the positive charge on ring carbon atoms to augment the activity profile, which can be achieved by attaching electronegative atoms to the ring carbon atoms. This observation is in tune with previously reported studies [14,32], which highlighted that the aromatic cavity of the S4 pocket of factor Xa is suitable for positively charged lipophilic moieties. Thus, QSAR provides consensus results with reported crystal structures of inhibitors for factor Xa. 

Another molecular descriptor with a positive correlation (R = 0.57) with activity is **aroN_sp2C_4B**, which represents the presence of an aromatic nitrogen atom within 4 bonds from sp^2^-hybridized carbon atoms. The positive coefficient indicates that higher the value of **aroN_sp2C_4B**, higher the activity for factor Xa. The molecular descriptors **aroN_sp2C_3B** and **aroN_sp2C_5B** represent the presence of aromatic nitrogen atoms within 3 and 5 bonds from sp^2^-hybridized carbon atoms, respectively. Interestingly, the binding affinity has slightly lower correlations with these two molecular descriptors (R = 0.55 and 0.54). Further, replacing **aroN_sp2C_4B** by **aroN_sp2C_5B** or **aroN_sp2C_3B** slightly reduced the performance of model with R^2^ = 0.79 and 0.80, respectively. Therefore, the optimum value of separation is 4 bonds.

**Figure 4:**
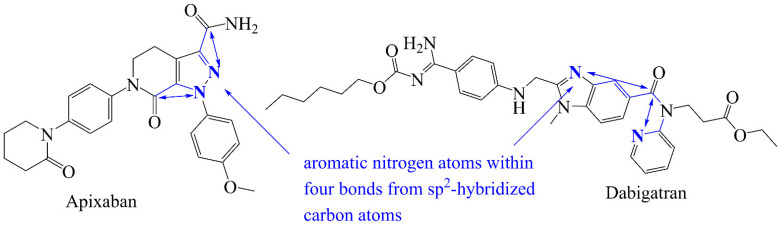
Marketed factor Xa inhibitors with **aroN_sp2C_4B** (blue colored).

From this descriptor, it also appears that the aromatic nitrogen atoms and sp^2^-hybridized carbon atoms could be individually able to augment activity. Therefore, we examined them individually by replacing **aroN_sp2C_4B** with **aroN** (number of aromatic nitrogen atoms) and then with **nsp2C** (number of sp^2^-hybridized carbon atoms) in the QSAR model, which resulted in R^2^ = 0.75 and 0.70, respectively. This decrease in the statistical performance of the model indicates that individually they are less useful. Moreover, **aroN** and **nsp2C** have a correlation of 0.54 and −0.16 with pKi respectively, which indicates that the presence of aromatic nitrogen atoms within four bonds from sp^2^-hybridized carbon atoms is required to have better activity. This observation is highlighted and supported by the presence of **aroN_sp2C_4B** in Apixaban. The X-ray-resolved structure of Apixaban with factor Xa confirmed that the pyrazole N2 nitrogen atom interacts with the backbone nitrogen atom of Gln192, whereas the carboxamide carbonyl makes a H-bond with NH of Gly216 [14,32]. Another example is Dabigatran (see Figure 4) [11,33]. 

**Figure 5:**
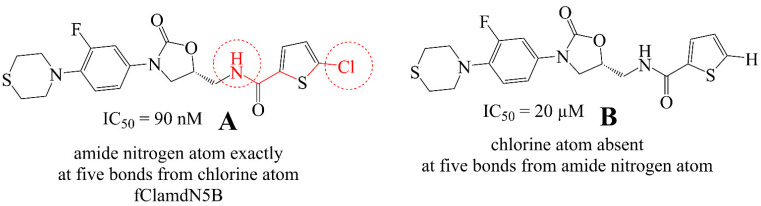
Representative examples molecule A and B to understand **fClamdN5B** (highlighted by red colored bonds and atoms)**.**

**fClamdN5B** signifies the frequency of the occurrence of an amide nitrogen atom exactly at five bonds from a chlorine atom. If the same amide nitrogen atom is simultaneously present at one to four bonds from any other chlorine atom, then it was excluded during the calculation of **fClamdN5B**. It has a positive coefficient in the developed QSAR model; therefore an increase in the value of this descriptor results in a better affinity for the target enzyme. In Figure 5, we have presented two examples, **A** and **B,** to understand the influence of **fClamdN5B**. The importance of **fClamdN5B** is vindicated by the fact that the NH of the chlorothiophene carboxamide of **A** is responsible for H-bond formation with Gly219 CO [14,32].

The molecular descriptor **fsp2Osp3O6B** stands for the frequency of occurrence of sp^3^-hybridized oxygen atoms exactly at six bonds from sp^2^-hybridized oxygen atoms. If the same sp^3^-hybridized oxygen atom is also present at five or less bonds from any other sp^2^-hybridized oxygen atom, then it was omitted during the calculation of **fsp2Osp3O6B**. Replacement of this molecular descriptor with a similar molecular descriptor **sp3O_sp2O_6B**, which represents the total number of sp^3^-hybridized oxygen atoms within 6 bonds from sp^2^-hybridized oxygen atoms, led to a visible decrease in the statistical performance of the model (R^2^ = 0.76). Therefore, the idea to exclude the same sp^3^-hybridized oxygen atom which is simultaneously present at five or less bonds from any other sp^2^-hybridized oxygen atom provided useful and additional understanding of concealed structural features.

The negative coefficient for **fsp2Osp3O6B** in the QSAR model indicates that increasing the value of this descriptor could lead to poor anti-thrombotic activity. In addition, it has a negative correlation of 0.43 with the activity. From 1121 molecules in the present data set, 358 molecules with a better activity (Ki = 0.007 to 10 nM) do not possess such a combination, whereas only 131 molecules have such a combination with their Ki ranging between 18,000 to 18.5 nM. Considering all these observations, it is reasonable to avoid such a combination of oxygen atoms to achieve a better activity profile.

The molecular descriptor **fsp2Nsp3O9B** signifies the frequency of occurrence of an sp^3^-hybridized oxygen atom exactly at nine bonds from sp^2^-hybridized nitrogen atom. If the same sp^3^-hybridized oxygen atom is also present at eight or less bonds from any other sp^2^-hybridized nitrogen atom then it was rejected during the calculation of **fsp2Nsp3O9B**. Replacing it with a very similar molecular descriptor **sp3O_sp2N_9B**, which counts the total number of sp^3^-hybridized oxygen atoms within nine bonds from a sp^2^-hybridized nitrogen atom, in the developed model led to a slightly poorer statistical performance (R^2^ = 0.78). Additionally, **fsp2Nsp3O9B** and **sp3O_sp2N_9B** have a correlation of −0.36 and −0.27 with activity values Ki, respectively. Clearly, **fsp2Nsp3O9B** is a better choice to be considered while predicting the activity. Thus, all these observations and its negative coefficient in the QSAR model indicate that lowering its value could lead to a better activity for factor Xa. 

Furthermore, the descriptor **fsp2Nsp3O9B** highlights the importance of the sp^2^-hybridized nitrogen atom, which in turn is present due to the presence of a guanidine group in majority of the compounds. Thus, this molecular descriptor indirectly identified the guanidine moiety as an important feature. In short, it indicates that a sp^3^-hybridized oxygen atom at exactly nine bonds from a sp^2^-hybridized nitrogen atom of a guanidine group should be avoided. Therefore, during the calculation of **fsp2Nsp3O9B,** the idea to reject the same sp^3^-hybridized oxygen atom, which is at the same time present at eight or less bonds from any other sp^2^-hybridized nitrogen atom, provided valuable and extended understanding of visually non-detectable structural features.

**fsp2Csp2O8B** represents the frequency of occurrence of sp^2^-hybridized oxygen atoms at exactly eight bonds from a sp^2^-hybridized carbon atom. If the same sp^2^-hybridized oxygen atom is concurrently present at seven or less bonds from any other sp^2^-hybridized carbon atom then it was rejected during the calculation of **fsp2Csp2O8B**. This molecular descriptor has a negative impact on a molecule’s anti-thrombotic activity profile, as it has a negative coefficient in the developed QSAR model. Therefore, the value of this molecular descriptor should be kept as low as possible.

From Figure 3a,b it is clear that the model is statistically robust, which is supported by a high value of R^2^_tr_ = 0.831 and a low value of RMSE_tr_ = 0.476_._ The compounds **46**, **59**, and **802** are outlier (see Figure 3c), probably chemicals with specific structural characteristics such as violation of Lipinski’s rule of five (molecular weight >500), higher number of electronegative elements (F, O, and N), stereocenters, and the presence of a pyrrolidine ring. The molecule **1109** appears as an outlier due to the presence of a good number of single bonds, which significantly enhances its flexibility and conformational space, thereby allowing it to adopt different shapes and conformations inside the active site of an enzyme. Principal component analysis (PCA) using QSARINS 2.2.4 [34] is available in the Supplementary Material (Figure S2). Y-randomization is a useful technique to identify chance correlations. For a good QSAR model, the value of R^2^_scr_ and Q^2^_scr_ should be low. Also, a graph between Kxy (correlation of the X block with response Y) and R^2^_scr_ and Q^2^_scr_ was plotted (see Figure 3d), which indicates that the QSAR model is free from chance correlations [34]. 

### 3.2. Comparison of QSAR Results with Reported Crystal Structures

**Figure 6:**
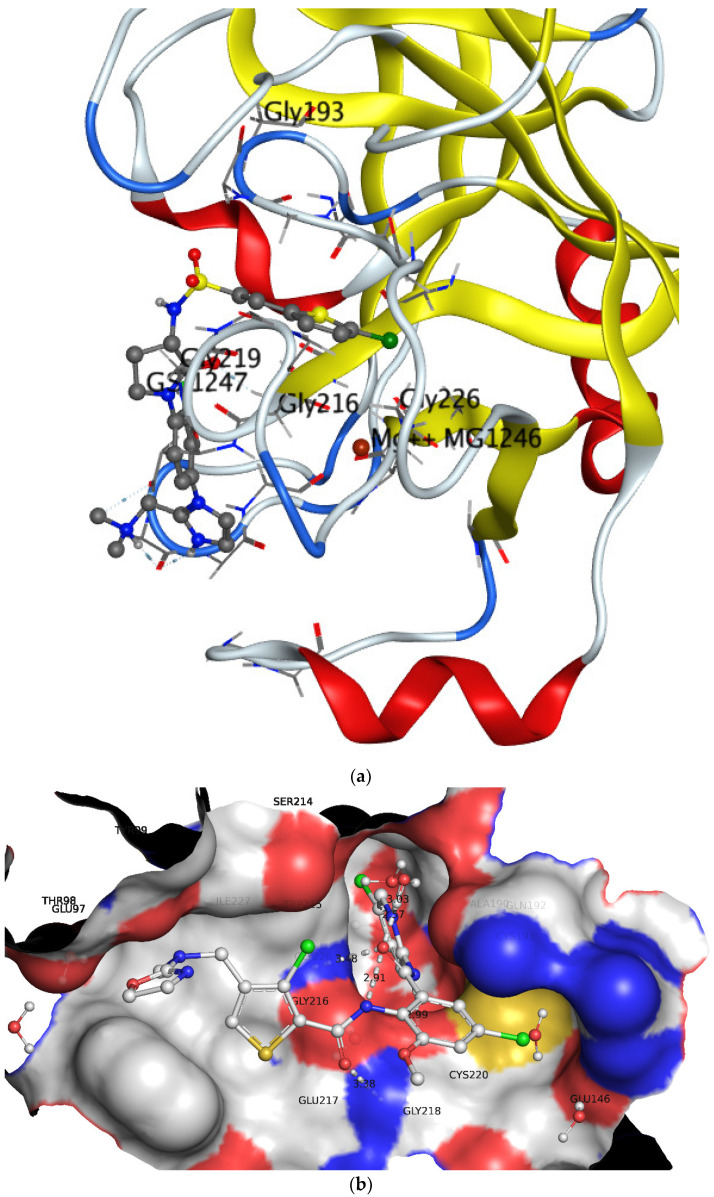
X-ray resolved pose for **1** in the active site of factor Xa (pdb 1MQ6) (**a**) without surface (**b**) with surface.

The active site of factor Xa consists of a catalytic triad of His57, Asp102 and Ser195 in the heavy chain along with two subsites S1 and S4 (see Figure 6) [14,32]. The S1 sub pocket is approximately 8Å deep and encompasses Trp215-Gly216 on one side and Ala190, Cys191 and Gln192 on the other side. A negatively charged Asp189 is present at the bottom of subsite S1 [11,13,14]. Consequently, S1 is a narrow pocket with substantial hydrophobic characters. Conversely, the S4 pocket is a relatively large lipophilic ‘U’ shaped pocket with Tyr99, Phe174 and Trp215 as gating residues responsible for its opening and closing [11,13,14]. 

**Figure 7:**
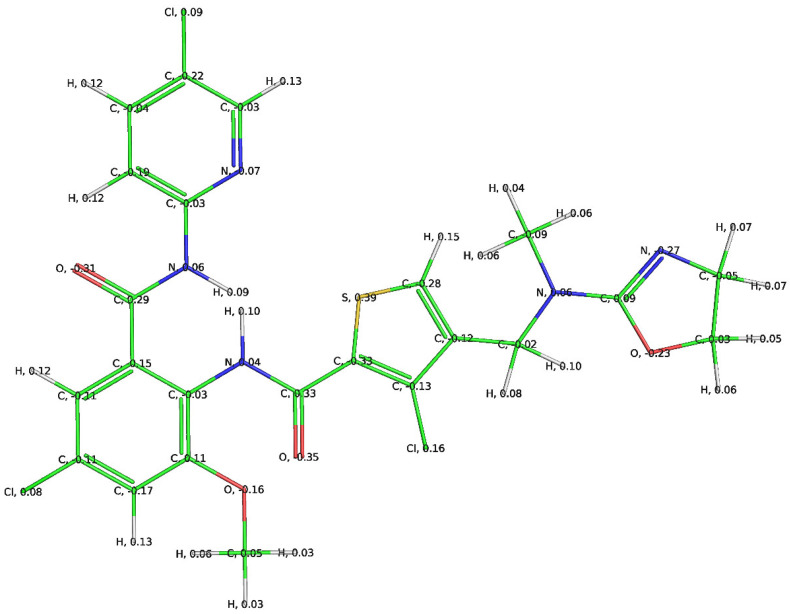
Compound **1** with partial charges in the active site of factor Xa (charges assigned using PM3 available in MOPAC2016 (http://openmopac.net)).

It is well-established that the S4 pocket is appropriate for lipophilic and positively charged moieties, thus making it highly suitable for developing a highly selective inhibitor. A comparison of QSAR results with the X-ray-resolved pose of 1 (pdb 1MQ6) in Figure 7 indicates that QSAR resulted in identification of consensus and complementary pharmacophoric features. Compound **1** has adopted a ‘J’ or ‘L’ shape conformation inside the active site of factor Xa with the presence of chloro-pyridine and oxazole moieties inside the S1 and S4 pockets, respectively. The chlorine atom of the chloro-pyridine moiety is responsible for lipophilic interactions with the nearby residues, whereas the amide nitrogen attached to the chloro-pyridine ring has established H-bonding with Gly218. This highlights the importance of chlorine and amide nitrogen atoms. The same combination is emphasized by QSAR analysis, as well. The oxazole moiety contains positively charged ring carbon atoms (see Figure 6) and interacts with residues of pocket S4, which is in tune with QSAR results. 

## 4. Materials and Methods

To build a thriving QSAR widely applicable model for anti-thrombosis activity, the following steps were sequentially performed: data collection and its curation, structure generation and calculation of molecular descriptors, objective feature selection (OFS), splitting the dataset into training and external validation sets, subjective feature selection, building a regression model and validation of developed model [16,17,19,26,27,28,35,36,37,38]. Thus, the present work follows the OECD recommended guidelines for the derivation of a QSAR model for factor Xa inhibitory activity.

### 4.1. Data Collection & Curation

**Figure 8:**
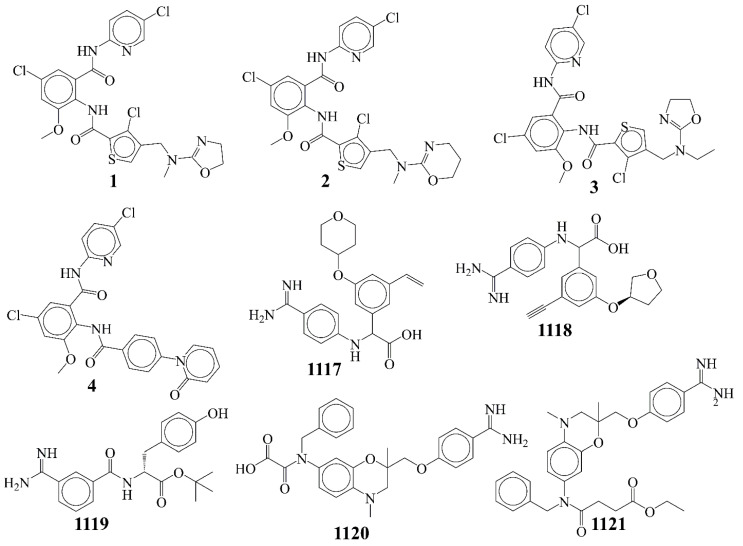
Representative examples from the selected dataset (four most active **1**–**4** and five least active **1117**–**1121** molecules).

The data set of factor Xa inhibitors used for building, training and validating the QSAR model was downloaded from ChEMBL (https://ebi.ac.uk/chembl/ accessed on 6 April 2021), which is a publicly available database. The data set comprises structurally diverse molecules experimentally tested for their activity for Factor Xa. Then, as a part of data curation, molecules with ambiguous enzyme inhibition constant (Ki) values, duplicates, salts, metal-based inhibitors, etc. were excluded [16,17,19,26,27,28,36,37,38]. Finally, the data set comprises diverse 1121 molecules with prodigious variation in structural scaffolds, which were tested experimentally for potency in terms of Ki (nM) (see the excel file ‘Supplementary Material-Final’ in the Supplementary Material). The experimental Ki values have ample variation between 0.007 to 18,000 nM. After that, Ki values were transformed to their negative logarithmic value (pKi = −log_10_Ki) so that a comparison of their values became easier. In Table 1 and Figure 8, some most and least active molecules have been included as examples only. 

### 4.2. Calculation of Molecular Descriptors and Objective Feature Selection (OFS)

The SMILES notations were transformed to 3D-optimized structures using Openbabel 3.1 [39] before calculation of molecular descriptors. The success of a QSAR analysis significantly depends on the appropriate calculation of diverse molecular descriptors to increase mechanistic interpretation, followed by their pruning to diminish the risk of overfitting from noisy redundant descriptors. To achieve these goals, PyDescriptor [40] was used to calculate more than 30,000 molecular descriptors. The vast pool of molecular descriptors comprises 1D- to 3D- molecular descriptors. Then, OFS was performed using QSARINS-2.2.4 [34] to eliminate near constant, constant and highly inter-correlated (|R| > 0.90) molecular descriptors. The final set contains 2682 molecular descriptors, which still comprise manifold descriptors leading to coverage of a broad descriptor space. 

### 4.3. Splitting the Data Set into Training and External Sets and Subjective Feature Selection (SFS)

**Figure 9:**
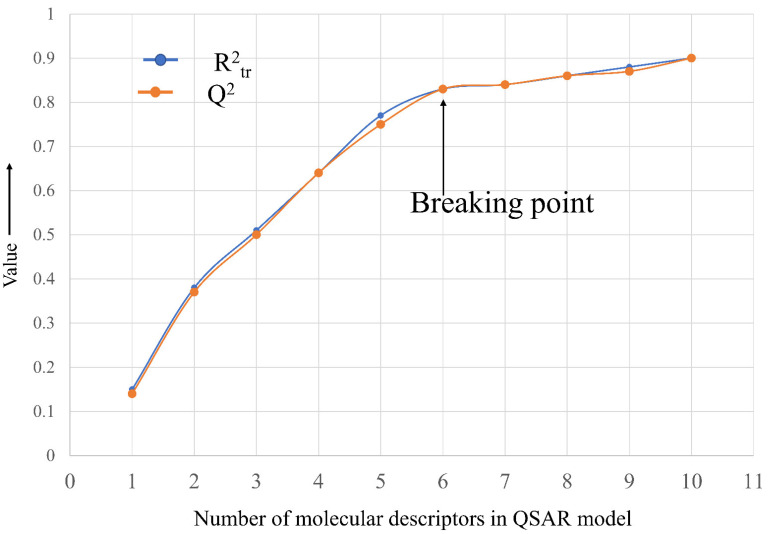
Plot of number of descriptors against coefficient of determination R^2^_tr_ and leave-one-out coefficient of determination Q^2^_LOO_ to identify the optimum number of descriptors.

Before exhaustive subjective feature selection, it is important to split the data set into training and prediction (also known as external or test set) sets with an appropriate composition and proportions to avoid information leakage [30]. To avoid any bias, the data set was randomly split into training (80% = 897 molecules) and prediction or external (20% = 224 molecules) sets. The sole purpose of a training set was to select an appropriate number of molecular descriptors, and the prediction/external set was used only for external validation of the model (Predictive QSAR). For subjective feature selection, the genetic algorithm unified with multilinear regression (GA-MLR) method implemented in QSARINS-2.2.4 was employed to choose relevant descriptors using Q^2^_LOO_ as a fitness parameter. An important step to develop a successful QSAR model with no over-fitting while maintaining acceptable interpretability is to have an adequate number of molecular descriptors in the model. In the present work, a graph (see Figure 9) was plotted between the number of molecular descriptors involved in the model and R^2^_tr_ and Q^2^_LOO_ values to obtain the so-called breaking point. Therefore, the number of molecular descriptors corresponding to the breaking point was considered optimum for model building. From Figure 9, it is clear that the breaking point corresponds to six variables. Therefore, QSAR models with more than six descriptors were rejected. 

### 4.4. Building Regression Model and Its Validation

A good QSAR model which has been properly validated using various methods such as cross-validation, external validation, Y-randomization and applicability domain (Williams plot) is useful for future utilization in virtual screening, molecular optimization, decision making, etc. The following statistical parameters and their recommended threshold values are routinely used to validate a model [19,23,24,27,41,42,43,44]: R^2^_tr_ ≥ 0.6, Q^2^_loo_ ≥ 0.5, Q^2^_LMO_ ≥ 0.6, R^2^ > Q^2^, R^2^_ex_ ≥ 0.6, RMSE_tr_ < RMSE_cv_, ΔK ≥ 0.05, CCC ≥ 0.80, Q^2^ − F^n^ ≥ 0.60, r^2^_m_ ≥ 0.5, (1 − r^2^/r_o_^2^) < 0.1, 0.9 ≤ k ≤ 1.1 or (1 − r^2^/r′_o_^2^) < 0.1, 0.9 ≤ k′ ≤ 1.1,| r_o_^2^ − r′_o_^2^| < 0.3, RMSE_ex,_ MAE_ex_, R^2^_ex_, Q^2^_F1_, Q^2^_F2_, Q^2^_F3_, and low R^2^_Yscr_, RMSE and MAE. The formulae for calculating these statistical parameters are available in the Supplementary Material. In addition, a Williams plot was plotted to evaluate the applicability domain of QSAR model.

## 5. Conclusions

In the present work, a six-descriptor-based and thoroughly validated GA–MLR QSAR model with R^2^_tr_ = 0.831, Q^2^_LMO_ = 0.828, and R^2^_ex_ = 0.783 was established to perceive the important pharmacophoric features that govern factor Xa inhibitory activity. As stated earlier, it is important to recognize prominent and visually unrecognizable pharmacophoric features associated with activity for factor Xa for different chemical classes. The QSAR analysis successfully identified a combination of reported and novel pharmacophoric features. The analysis vindicates that chlorine with amide nitrogen atoms, the sum of partial charges on positively charged ring carbon atoms, the importance of aromatic nitrogen and sp^2^-hybridized carbon atoms, etc. are prominent features to be retained in future optimizations. Conversely, new structural features such as the combination of sp^3^-hybridized oxygen atoms at exactly six bonds from sp^2^-hybridized oxygen atoms, sp^3^-hybridized oxygen atoms at exactly nine bonds from sp^2^-hybridized nitrogen atoms, and sp^2^-hybridized oxygen atoms at exactly eight bonds from sp^2^-hybridized carbon atoms should be avoided to have a better activity profile against factor Xa. The QSAR model has a good balance of predictive ability and mechanistic interpretations, which are further supported by the reported crystal structures of factor Xa inhibitors.

## Figures and Tables

**Table 1:** SMILES notation, Ki (nM) and pKi (M) of five most and least active molecules of the selected data set.

**S.N.**	**SMILES Notation**	**Ki** **(nM)**	**pKi** **(M)**
1	COc1cc(Cl)cc(C(=O)Nc2ccc(Cl)cn2)c1NC(=O)c1scc(CN(C)C2=NCCO2)c1Cl	0.007	11.155
2	COc1cc(Cl)cc(C(=O)Nc2ccc(Cl)cn2)c1NC(=O)c1scc(CN(C)C2=NCCCO2)c1Cl	0.012	10.921
3	CCN(Cc1csc(C(=O)Nc2c(OC)cc(Cl)cc2C(=O)Nc2ccc(Cl)cn2)c1Cl)C1=NCCO1	0.012	10.921
4	COc1cc(Cl)cc(C(=O)Nc2ccc(Cl)cn2)c1NC(=O)c1ccc(-n2ccccc2=O)cc1	0.013	10.886
5	COc1cc(Cl)cc(C(=O)Nc2ccc(Cl)cn2)c1NC(=O)c1scc(CN(C)C2=NCCS2)c1Cl	0.024	10.62
1117	C=Cc1cc(OC2CCOCC2)cc(C(Nc2ccc(C(=N)N)cc2)C(=O)O)c1	13,300	4.876
1118	C#Cc1cc(O[C@@H]2CCOC2)cc(C(Nc2ccc(C(=N)N)cc2)C(=O)O)c1	15,300	4.815
1119	CC(C)(C)OC(=O)[C@@H](Cc1ccc(O)cc1)NC(=O)c1cccc(C(=N)N)c1	16,000	4.796
1120	CN1CC(C)(COc2ccc(C(=N)N)cc2)Oc2cc(N(Cc3ccccc3)C(=O)C(=O)O)ccc21	16,600	4.78
1121	CCOC(=O)CCC(=O)N(Cc1ccccc1)c1ccc2c(c1)OC(C)(COc1ccc(C(=N)N)cc1)CN2C	18,000	4.745

## Data Availability

The data is available in the Supplementary Section.

## Supplementary Materials

The following are available online at https://www.mdpi.com/article/10.3390/ijms22158352/s1.

## Abbreviations

SMILES	Simplified molecular-input line-entry system
GA	Genetic algorithm
MLR	Multiple linear regression
QSAR	Quantitative structure−activity relationship
WHO	World Health Organization
ADMET	Absorption, distribution, metabolism, excretion, and toxicity
OLS	Ordinary least square
QSARINS	QSAR Insubria
OECD	Organisation for Economic Co-operation and Development
