# Supplementary material for: Mechanistic and Predictive QSAR Analysis of Diverse Molecules to Capture Salient and Hidden Pharmacophores for Anti-Thrombotic Activity
Source: Int J Mol Sci. 2021 Aug 3;22(15):8352. doi: 10.3390/ijms22158352 (PMC8348508; doi:10.3390/ijms22158352)
Supplement: Supplementary file 1 [file ijms-22-08352-s001.zip › ijms-1284074-supplementary.pdf]

Figure S1: Score plot after PCA using QSARINS 2.2.4.

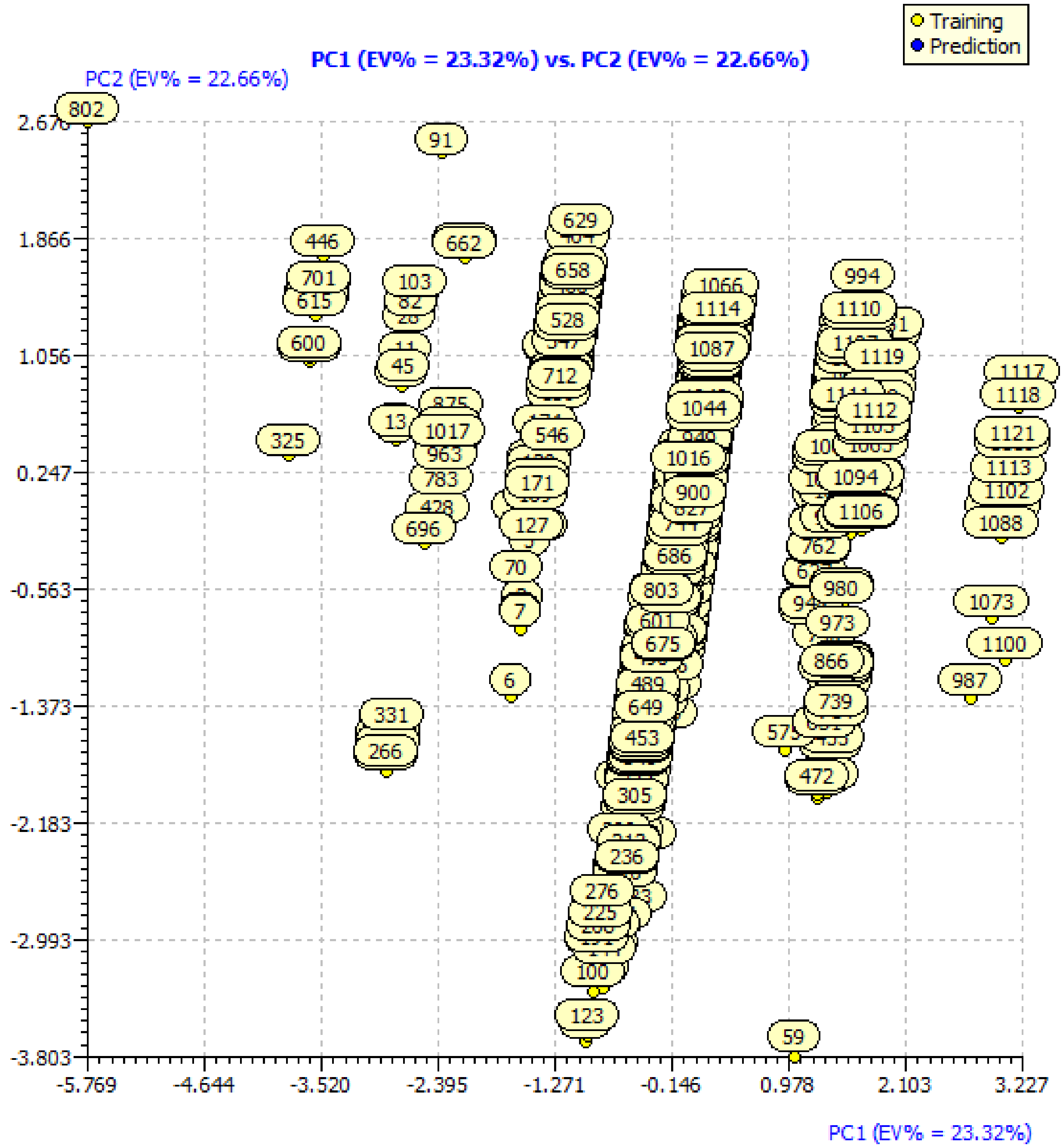

Figure S2: Loading plot after PCA using QSARINS 2.2.4.

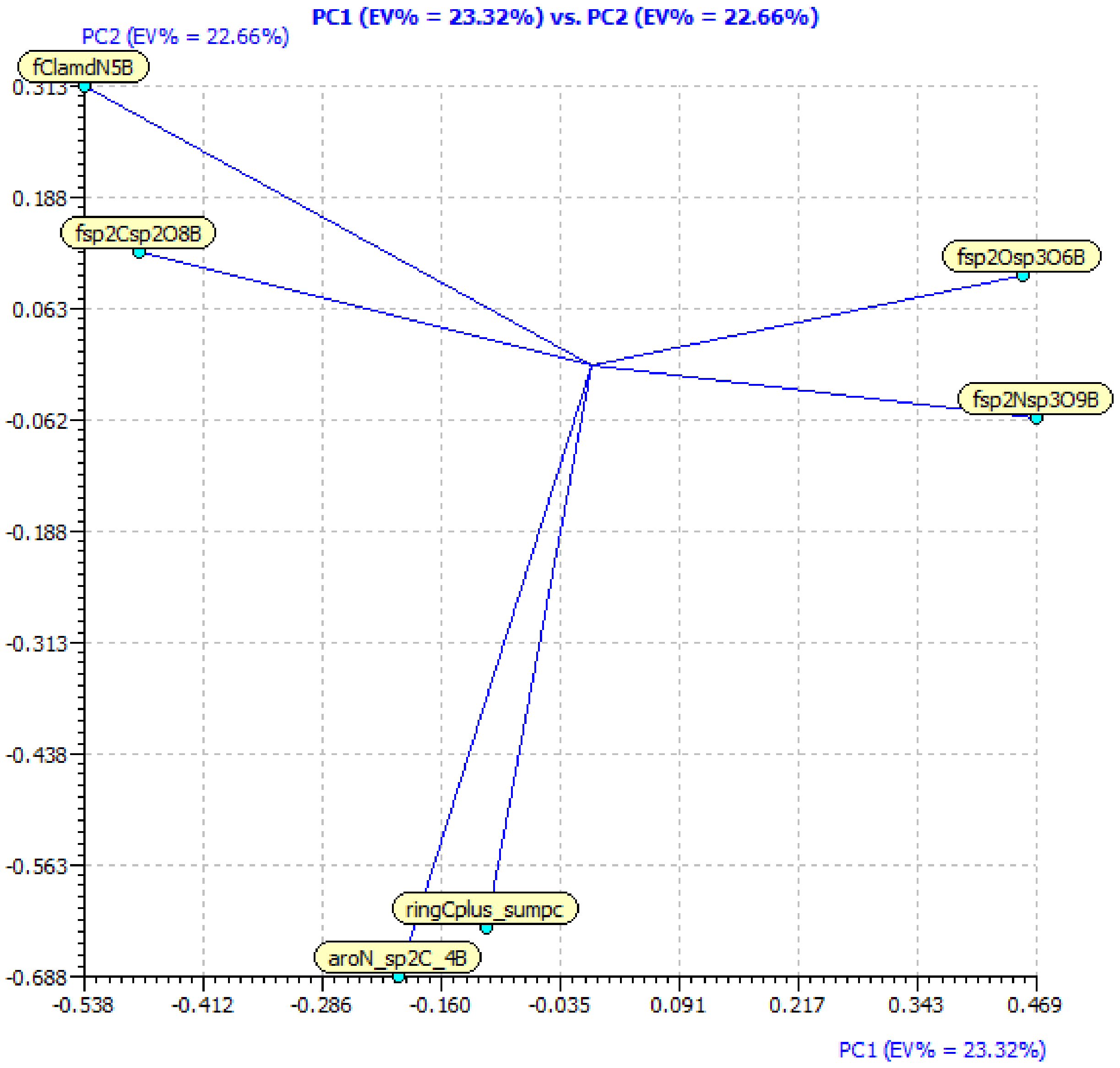

Table S1: Correlation matrix for molecular descriptors used in the present work.

	ringCplus_sumpc	aroN_sp2C_4B	fClamdN5B	fsp2Osp3O6B	fsp2Nsp3O9B	fsp2Csp2O8B
ringCplus_sumpc	1.0000					
aroN_sp2C_4B	0.3276	1.0000				
fClamdN5B	-0.0310	-0.0919	1.0000			
fsp2Osp3O6B	-0.0023	-0.1230	-0.1140	1.0000		
fsp2Nsp3O9B	0.1461	-0.1462	-0.1224	0.1690	1.0000	
fsp2Csp2O8B	0.1302	-0.0641	0.2671	-0.0338	-0.0648	1.0000

Different validation parameters for model-1:

R^2_{tr} = 0.831, $R^2_{adj.}$ = 0.83, LOF = 0.233, Kxx = 0.188, ΔK = 0.07, $RMSE_{tr}$ = 0.476, MAE_{tr} = 0.38, RSS_{tr} = 203.239, CCC_{tr} = 0.908, s = 0.478, F = 731.048, R^2_{cv} (Q^2loo) = 0.829, $RMSE_{cv}$ = 0.479, MAE_{cv} = 0.383, $PRESS_{cv}$ = 206.131, CCC_{cv} = 0.907, Q^2_{LMO} = 0.828, R^2_{Yscr} = 0.007, Q^2_{Yscr} = -0.009, $RMSE_{ex}$ = 0.526, MAE_{ex} = 0.417, $PRESS_{ext}$ = 61.945, R^2_{ex} = 0.783, Q^2-F^1 = 0.782, Q^2-F^2 = 0.782, Q^2-F^3 = 0.794, CCC_{ex} = 0.874, R^2-ExPy = 0.783, R'^2_o = 0.704, k' = 0.996, 1-(R^2/ R'^2_o) = 0.101, r'^2m = 0.563, R_o^2 = 0.782, k = 0.999, 1-(R^2-ExPy/ R_o^2) = 0.001, r^2m = 0.76
